# Impact of corticosteroid administration in septic shock on glycemic variability

**DOI:** 10.1186/cc13635

**Published:** 2014-03-17

**Authors:** L Mirea, R Ungureanu, D Pavelescu, IC Grintescu, C Dumitrache, D Mirea, I Grintescu

**Affiliations:** 1 Clinical Emergency Hospital of Bucharest, Romania; 2 National Institute of Endocrinology, Bucharest, Romania; 3 Elias University Emergency Hospital, Bucharest, Romania

## Introduction

The purpose of this study was to assess the relation between glycemic control and the severity of sepsis in a cohort of patients with vasopressor-dependent septic shock treated with corticosteroids [1,2]

## Methods

In a prospective, controlled study, 134 patients with septic shock were randomized into three study groups: group A *(n *= 43), 200 mg/day hydrocortisone hemisuccinate in four daily doses; group B (*n *= 47), same dose of hydrocortisone hemisuccinate in continuous administration; group C (*n *= 44), no hydrocortisone hemisuccinate. Patients with diabetes mellitus were excluded. The duration of hydrocortisone treatment was a maximum 7 days. The target blood glucose (BG) level was below 180 mg/dl. BG values were analyzed by calculating mean daily values, standard deviation (SD) of BG values as an index of glycemic variability, and insulin doses. The local ethics committee approved the study.

## Results

There were no differences between the three groups at the beginning of the study regarding demographic data and the clinical characteristics, including BG value. BG levels were strongly correlated with severity of septic shock estimated by APACHE II score (*r *= +0.241; *P *= 0.005) or Simplified Acute Physiology Score II (*r *= 0.280; *P *= 0.001) - Pearson correlation. The risk of death is significantly increased if SD of BG is more than 20 mg/dl (67.7% vs. 20.8%, *P *= 0.000). A total 94.4% of deceased patients in group A registered a SD of BG more than 20 mg/dl versus 89.5% in group B or 40% in group C (*P *= 0.006). In total, 53.5% of patients in group A needed insulin therapy versus 25.5% in group B or 27.3% in group C. The dose was between 30.28 ± 6.65 UI/day in group A, 37.85 ± 11.95 UI/day in group B, and 14.28 ± 5.76 UI/day in group C *(P *>0.05).

## Conclusion

BG variability is highly associated with mortality compared with BG mean daily value or insulin dose. SD levels above 20 mg/dl were associated with a significantly higher mortality rate relative to those with SD levels below 20 mg/dl.
